# Valedictory Addresses

**Published:** 1849-05

**Authors:** 


### VALEDICTORY ADDRESSES.

We are glad to perceive a growing disposition in medical classes to publish the valedictory addresses of their teachers. The sentiments uttered on the occasion of the last formal meeting of pupils and professors ought to be such as are worth treasuring up, and the effect of giving them to the public in an enduring form will be to elevate the character of such discourses. In those which have come under our eye this season we find much to applaud. Their tone is excellent, and they embody a code of professional morals which ought to meet with universal acceptance. Prof. Harrison, of Cincinnati, addressed his young friends "on the sources and benefits of Professional Earnestness," a fine example of which is afforded by every page of his eloquent discourse. The address of Prof. Barbour, of St. Louis, is in a kindred style, full of noble sentiments, clothed in forcible language. Our brethren of the College of Dental Surgery, at Baltimore, are represented in this line by Dr. C. O. Cone, who pronounced an appropriate and instructive discourse to the pupils of that institution, which has been published. All these addresses afford indications of progress, past and to come. They show that vast improvements have been made, and give promise in their tone of greater improvements in time to come.

